# Magnetite-Doped Activated Carbon Beads and Powder Derived from Chitosan for Adsorption of Emerging Contaminants in Drinkable Water

**DOI:** 10.3390/molecules30224443

**Published:** 2025-11-18

**Authors:** Nirav P. Raval, Laurence Reinert, Laurent Duclaux, Nathalie Cottin, Noriko Yoshizawa, Jimmy Nicolle, Anandu Chandran, Fabrice Muller

**Affiliations:** 1 Environnements, Dynamiques et Territoires de la Montagnes (EDYTEM), Université Savoie Mont Blanc, 73000 Chambéry, France; laurence.reinert@univ-smb.fr (L.R.);; 2 Department of Environmental Science and Engineering, School of Engineering and Sciences, SRM University-*AP*, Amaravati 522 240, Andhra Pradesh, India; niravkumar.r@srmap.edu.in (N.P.R.);; 3 National Institute of Advanced Industrial Science and Technology (AIST), Research Planning Office of Zero Emission, 1-1-1 Umezono, Tsukuba 305-8560, Ibaraki, Japan; noriko-yoshizawa@aist.go.jp; 4 Interfaces, Confinement, Matériaux et Nanostructures (ICMN), CNRS-Université d’Orléans, UMR 7374, 1b rue de la Férollerie, CS 40059, 45071 Orléans Cedex 2, France; jimmy.nicolle@cnrs-orleans.fr (J.N.);

**Keywords:** chitosan, carbon beads, emerging contaminants, mixture toxicity, human health

## Abstract

Activated carbon beads, some of which contain Fe_3_O_4_ nanoparticles or graphene oxide, were synthesized by thermal activation (700 °C) of chitosan hydrogel beads. Materials showed a multiporous scale (micro/meso/macro) and BET specific surface areas in the 260–572 m^2^.g^−1^ range. The adsorption kinetics of beads and powders resulting from their grinding were studied for a mixture of six micropollutants (bisphenol A, carbofuran, carbamazepine, diclofenac, dimethoate and imidacloprid) dissolved in spring water. While the adsorption kinetics on the beads (pH 7.3, 25 °C, 10–100 µg.L^−1^) are slow (equilibrium time > 24 h), the powdered samples are more efficient: for an initial concentration of 50 μg.L^−1^ of each pollutant (0.1 g.L^−1^ of adsorbent), 50 to 99% of the micropollutants introduced into the solution were removed after 4 h of contact time. Depending on the pollutant nature, the adsorption isotherms (0.2–40 μg.L^−1^) studied for an activated carbon powder containing Fe_3_O_4_ (1 mass %) are either of Langmuir or Freundlich type, or they follow Henry’s law and are related to the different properties of the molecules.

## 1. Introduction

Access to clean, potable water and basic sanitation facilities is recognized as a fundamental human right [1]. However, in the Anthropocene, >2 billion people consume water contaminated with fecal matter, ~4.5 billion lack adequate sanitation, and 842,000 water, sanitation and hygiene (WASH)-related deaths have been reported from unsafe drinking water [2,3,4]. More than half of the global population (~4 billion) experiences severe water scarcity for at least one month per year, predominantly in India (1 billion) and China (0.9 billion), with 130 million affected in the United States [5,6,7,8]. With the global population projected to reach 9.7 billion by 2050, potable water demand is expected to increase by 55% [9,10,11]. In this context, wastewater reuse has emerged as a promising solution, providing a ‘new’ source of clean water and supporting the sixth Sustainable Development Goal (SDG-6) by restoring aquatic ecosystems and mitigating pollution in natural water bodies [12,13,14,15,16].

Rapid urbanization, population growth and modern lifestyles have driven unprecedented industrialization, leading to the global proliferation of multiple emerging contaminants (MECs) [17,18,19]. These contaminants adversely affect biotic and abiotic indicators, compromise human immune responses and can act as potential infection agents. While some developed nations, such as Germany, Belgium, the Netherlands, France and Denmark, have proactively instituted policies and regulatory measures to address ECs, many developing countries lack such guidelines [20,21,22,23]. MECs typically occur in trace amounts (ng.L^−1^ to µg.L^−1^) and are difficult to remove with conventional treatment, with the added concern of promoting resistant bacteria and viruses (superbugs) [24,25,26].

To address the presence of contaminants in wastewater, activated carbon (AC)-supported wastewater treatment is widely practiced globally, especially at the tertiary stage [27]. This method functions as a multibarrier treatment process designed to eliminate ECs, ensuring the achievement of direct potable reuse (DPR) with essential reliability, redundancy, robustness and resilience (4Rs) [28,29]. However, commercial AC is costly, produced from limited carbon sources (coal, petroleum coke, peat) and contributes indirectly to energy demand [30,31,32]. To tackle this economic and environmental challenge, researchers have diligently worked towards producing ACs from various precursors, employing both physical and chemical activation methods. These precursors encompass lignocellulosic biomasses such as coconut shell, paddy straw, rice husk, teff husk, etc. [33,34,35], microbial biomasses [36], municipal/industrial waste materials including sludge [37,38] and synthetic/natural polymers [39]. Despite progress, these methods are often time-consuming, involve multiple activation steps and may introduce secondary contaminants [40].

In response to the aforementioned challenges, the research community has delved into the utilization of natural biopolymeric materials for the production of AC. Notably, AC is commonly formulated as a composite material with various biopolymers, including alginate [41,42], cellulose [43] and chitosan (CS) [44], to address the removal of a spectrum of contaminants ranging from legacy to emerging pollutants. However, in the synthesis of such adsorbents, the carbon derived from natural materials is impregnated within the biopolymeric hydrogel [45,46]. Consequently, this incorporation diminishes the overall surface area of the carbon, resulting in lower removal efficiency [47]. To overcome this, direct carbonization of biopolymers via chemical activation (e.g., NaOH [47], KOH [48],) has been employed, producing well-developed pore structures with high surface areas. Remarkably, despite the enhanced surface properties of these meticulously engineered materials, their application for the adsorption of ECs remains unexplored.

Recent advances also highlight the use of metal-oxide nanoparticles to enhance adsorption, particularly when doping carbon adsorbents. Iron (Fe)-based nanoparticles, especially magnetite, impart magnetic properties, create active sites, exhibit low toxicity and enable efficient pollutant removal [49,50,51,52,53]. Several studies have demonstrated the effectiveness of such carbon–iron nanocomposites in water treatment applications. For instance, Efficient removal of six emerging contaminants (triclosan, bisphenol-A, tonalide^®^, metolachlor, ketoprofen, and estriol) was achieved using PVP-coated magnetite nanoparticles supported on granular AC, with bisphenol-A (98%) and ketoprofen (95%) showing the highest removal efficiencies using only 0.1 mg of adsorbent within 15 min [54]. Subsequent studies demonstrated the effective remediation of tetracycline (66.4%) and paracetamol (71.6%) using iron-doped carbon adsorbents derived from pine fruit waste [55]. A magnetic nanosorbent (AC/Fe_3_O_4_) was synthesized by modifying powdered AC with magnetite nanoparticles and applied for magnetic solid-phase extraction of BPA and 17α-ethinylestradiol from water samples [56]. Co-doping iron with sulfidation in AC derived from coconut shell precursors enhanced the adsorption of the hydrophobic contaminant triclosan [57]. Additionally, Fe-loaded AC synthesized from Macauba palm achieved up to 93% amoxicillin removal through an adsorption/photocatalytic oxidation process [58]. However, these studies typically focus on specific contaminants, lack systematic synthesis optimization and rarely explore CS-derived carbons produced via cost-effective, non-toxic gelation methods that combine biopolymer sustainability with magnetic recoverability.

To bridge this gap, this study aims to optimize the synthesis of three different CS carbon beads (i.e., C-Cs, C-CsF and C-CsG) and evaluate their application for MEC removal from simulated wastewater. The specific objectives are as follows: (i) to optimize fabrication of Fe-doped CS carbon beads, utilizing NaOH gelation under variable conditions such as temperature and time; (ii) to characterize the synthesized carbons using advanced techniques to assess their adsorption potential; and (iii) to conduct kinetic and equilibrium adsorption studies to determine removal rates and maximum adsorption capacities for MECs.

## 2. Experimental Section

### 2.1. Chemicals and Reagents

Shrimp-shells-derived CS power was procured from Mahtani Chitosan PVT. Ltd., (Veraval, Gujarat, India, batch no. 342). Reagent-grade acetic acid (CH_3_COOH, ≥99.7%), used to formulate a viscous CS solution, and sodium hydroxide (NaOH, >98%), employed to gelatinize the CS solution, were sourced from Merck-Sigma-Aldrich (Darmstadt, Germany). For the synthesis of magnetite nanoparticles (Fe_3_O_4_), the precursor salts of Fe^2+^ (FeCl_2_.4H_2_O, ≥98%) and Fe^3+^ (FeCl_3_.6H_2_O, ≥98%) were acquired from Carl Roth (Lauterbourg, France). Deionized water was utilized for the preparation of acidic solutions of CS, the NaOH basic bath and for the dissolution of the iron salts. Analytical-grade reagents were employed throughout the study, except where specifically indicated.

Bisphenol-A (BPA), carbofuran (CBF), carbamazepine (CBZ), dimethoate (DMA), diclofenac (DCF), imidacloprid (ICP), high-performance liquid chromatography (HPLC) grade methanol (≥99.8%) and acetonitrile (≥99.8%) were purchased from Merck (Darmstadt, Germany). Table 1 provides a comprehensive overview of the detailed physicochemical characteristics of the selected ECs. Different stock solutions of BPA, CBF, ICP and DMA (100 mg.L^−1^), CBZ (10 mg.L^−1^) and DCF (1.5 mg.L^−1^) were prepared in Auvergne Mountain spring water (source: Grand Barbier, Varennes-sur-Allier, France) and have a pH value of 7.3. The standard solutions of the mixture of the 6 micropollutants, ranging in concentration from 10 to 250 µg.L^−1^ (for each contaminant in the mixture), were meticulously prepared by suitably mixing and diluting the stock solutions of the selected ECs.

### 2.2. Synthesis Procedures

#### 2.2.1. Synthesis of Oleic Acid Coated Fe_3_O_4_ Nanoparticles

Magnetic nanoparticles coated with multilayer oleic acid were synthesized via a chemical co-precipitation method, following the methodology previously documented in [59]. Specifically, solutions of FeCl_3_.6H_2_O (0.34 mol.L^−1^) and FeCl_2_.4H_2_O (0.17 mol.L^−1^) were vigorously mixed and stirred at 500 rpm to achieve homogeneity. Subsequently, the solution was heated for 1 h at a temperature range of 80 °C. Upon attaining a colloidal dark yellow hue, 20 mL of ammonia solution (30%) was added drop-wise until black precipitates formed. Further, oleic acid (10 mL) was added drop-wise to create a protective layer around the nanoparticles, preventing agglomeration attributed to their high surface energy. The resulting precipitates underwent meticulous washing with acetone to eliminate unreacted residues of oleic acid and enhance the magnetization properties. The final product was dried and stored in an airtight container for subsequent doping experiments.

#### 2.2.2. Synthesis of NaOH Impregnated Hydrogel Beads of Pure CS, Fe-Doped CS and CS/GO

Three kinds of beads were prepared by gelification of different solutions and suspensions: pure CS beads, Fe-doped CS beads and CS/GO hybrid beads (Table 2).

The viscous CS solutions (5 mass. %) were prepared by dissolving the appropriate amount of CS powder into 0.5 mol.L^−1^ CH_3_COOH solution. The CS solutions were kept under agitation in a shaker at 200 rpm until complete dissolution and used for pure CS bead preparation.

For the preparation of Fe-doped CS beads, a weighted amount (0.05–0.25 g) of coated magnetic nanoparticles was added to the obtained CS solution (100 mL) and sonicated for 15 min at 35 °C (in ultrasonic bath, 38 kHz), followed by agitation to obtain the complete homogenization prior to the gelification step.

50 mL of graphene oxide (GO) solution (concentration 4.5 g.L^−1^) was added to the pure CS solution in acetic acid (50 mL at 5 mass. % CS) in order to obtain CS/GO hybrid beads. The viscous mixtures were stirred for 30 min, sonicated at 45 °C for 15 min in an ultrasonic bath (38 kHz) and again stirred for 30 min.

The gelatinous solution (pure CS in acetic acid solution) or the dispersion of magnetic particles and/or GO in CS solutions was then drop-wise introduced into a known concentration (0.25 mol.L^−1^) of NaOH gelation solution to obtain the NaOH impregnation ratio (9%) with respect to the CS weight. For the projection of viscous dispersion (CS solution + magnetic particles or CS solution + GO) or pure CS solution into the gelation bath, a peristaltic pump operated at a flow rate of 50 mL.h^−1^ and 2.0 mm diameter tubing attached with dispensing needle (RS, France, KDS1812P, 12.7 mm length, 1.27 outside diameter and 0.97 mm internal diameter) were used. The projection of the CS solution into the gelation bath was performed from a 25 cm height to prevent any possible bubble/tail formation. After the contact with the alkaline solution, the generated spherical-shaped CS-based beads were agitated for a predefined time period of 2 h, sieved and drained before the pyrolytic activation.

#### 2.2.3. Activation of CS-Based Beads

The drained hydrogel beads were placed into a cylindrical alumina crucible (Anderman Céramiques, Olivet, France; EA998, ID: T103, capacity: 60 mL, height: 60 mm and diameter: 40 mm). The beads filled crucible was placed in the center of the large-size crucible (Anderman Céramiques, EA998, ID: T105, capacity: 250 mL, height: 100 mm and diameter: 65 mm) and covered with coke to provide the inert condition. Finally, the crucible was placed inside the preheated muffle furnace (Nabertherm, Lilienthal, Germany; L(T) 9/11) at different temperatures (600–1000 °C) for 1 h. Afterwards, the obtained carbon beads were washed multiple times with DIW to bring the solution pH in the neutral range (6.5 to 7.0), dried in a hot air oven at 80 °C for 24 h and characterized to understand their physicochemical properties.

Three types of pyrolyzed carbon beads were obtained: AC beads from pure CS, AC/iron oxide beads and AC/GO beads, referred to as C-Cs, C-CsF and C-CsG, respectively. The conditions of preparation of the AC beads and their compositions are summarized in Table 2. Further in the article, the C-Cs beads that underwent pyrolysis at a T temperature (expressed in °C) are labeled C-Cs-T. Namely, C-Cs-700 AC beads have been prepared by heat treatment at 700 °C.

### 2.3. Characterization of the AC-Based Beads

To verify the porous structure of the synthesized AC adsorbents, N_2_ adsorption/desorption isotherms were obtained by initially degassing the samples at 100 °C for at least 12 h, followed by analysis at 196 °C using a Brunauer–Emmett–Teller (BET) sorptometer (Micromeritics ASAP 2020, Norcross, GA, USA). For microporous materials, the BET surface area (S_BET_) was deduced by analyzing the N_2_ adsorption isotherm in the relative pressure range of 0.01–0.05. The total pore volume (micro and mesopore volumes) was determined with the MicroActive software, Version 5.02, from the N_2_ adsorbed amount at P/P_0_ = 0.995, assuming that liquid nitrogen at this pressure fills all the pores. The microporous volume was determined by using the Dubinin–Radushkevich model in the relative pressure range of 0.01–0.1. This model is based on the Polanyi adsorption potential theory and is appropriate for characterizing micropores in carbonaceous adsorbents. The mesopore volume was subsequently obtained by subtracting the micropore volume from the total pore volume. The pore size distributions were obtained from the BJH (Barrett Joiner Halenda) model applied to N_2_ adsorption isotherms at 77 K, assuming cylindrical shapes of the pores and a Hasley equation to calculate the thickness of adsorbed layers.

The X-ray Diffractometer (XRD) (D8 Advance, Brucker AXS, Karlsruhe, Germany) operated with Cu-Kα radiation (λ = 1.54051 Å) at 40 kV and 40 mA was employed to record the XRD diffractogram of the synthesized materials within the 2θ range from 5° to 70° (scan rate of 5°/min). Morphological and compositional characterization of the surfaces of the C-Cs-700 (heat treated at 700 °C) and C-CsF/2% beads was performed using a FEG-SEM (Field Emission Gun Scanning Electron Microscope, ZEISS ULTRA 55 Gemini, ZEISS, Oberkochen, Germany) coupled with an EDS (Energy Dispersive Spectrometry) equipped with a Silicon Drift Detector (BRUKER AXS-30 mm^2^, Bruker AXS Microanalysis, Berlin, Germany) X-ray Energy Dispersive spectrometer. Prior to observation, the samples were coated with an amorphous carbon deposit obtained by cathodic evaporation.

TEM (Transmission Electron Microscope, JEOL EM-2100F, JEOL, Akishima, Japan) observations of the C-Cs (heat treated at 600–1000 °C), C-CsF/2%, C-CsF/5% and C-CsG beads were carried out at an accelerating voltage of 200 kV. For the TEM, the samples were dispersed on a copper microgrid after being ground in an agate mortar.

X-ray Photoelectron Spectroscopy (XPS) was used to examine the surface composition of the C-CsF fabricated carbon beads (1–5 mass. % of iron oxide) decorated with iron oxide. XPS analysis has been carried out using a Thermofischer Escalab Xi system (Thermofischer Scientific Ltd., East Grinstead, UK) operating under 10^−10^ mbars, with a spot size of 600 μm. The spectrometer is equipped with a non-monochromatic dual anode X-ray source providing AlKα or Ag Lα photons of energy of 1486.6 eV or 3000 eV. Unmonochromatic AlKα X-rays (hν = 1486.6 eV) have been used to irradiate the investigated samples. A background, based on the Shirley iterative method, has been used to subtract the inelastic background to all the XPS spectra.

The surface charge of some synthesized carbon beads doped with magnetite (1 mass. %) was investigated by measuring the isoelectric pH using the three-point calibrated benchtop JENWAY 3510 standard digital pH meter (Cole-Parmer, St Neots, UK) with an accuracy of ±0.03. In brief, 10 mL suspensions of 10 mg of ground solid samples were prepared and adjusted to different pH values ranging from 2.0 to 12.0 using 0.01 mol.L^−1^ NaOH or 0.01 mol.L^−1^ HCl. The zeta potential of the suspensions was then measured using a Litesizer™ 500 particle analyzer (Anton Paar, Graz, Austria). The isoelectric pH point of the material is obtained when the zeta potential is zero.

### 2.4. Adsorption Experiment for Mixture of ECs Removal

To generate the semi-scale field conditions, the batch kinetics experiments were conducted by agitating 1 L of an ECs mixture solution made in spring water (source: Grand Barbier, France) with 0.1 g chitosan–carbon beads at 298 K, 175 rpm in an incubator shaker (New Brunswick Innova^®^44, Eppendorf, Hamburg, Germany) for 24 h. The adsorption kinetics of the ECs in the solution mixture were first investigated on the entire carbon beads C-Cs-700, C-CsF/1% and C-CsG. The initial concentrations of BPA, CBF, ICP and DMA were set to Ci = 10 µg.L^−1^, while the initial concentrations of CBZ and DCF were set to Ci = 100 µg.L^−1^. The portion of the samples at the predefined time intervals was collected and analyzed (see below) to identify the concentration of the ECs.

Secondly, the adsorption kinetics of all the ECs on the C-CsF powder was studied using an equi-massic mixture solution for each EC, with the same initial concentration for each of the targeted ECs set at Ci = 50 µg.L^−1^. The powder of C-CsF/1% was obtained by milling the beads in an agate mortar. For the kinetic studies, at regular time intervals, 1 mL of solution was taken out with a glass syringe of 5 mL capacity, filtered using 1.2 μm, Ø 25 mm glass filters (Whatman GF/C) and collected into 1.5 mL amber glass chromatography vials (32 × 11.6 mm; Chromoptic, Villejust, France).

In order to obtain the adsorption isotherm of each EC in the mixture, the initial concentration of the mixture solution was varied within the range of 10 to 250 µg.L^−1^. The solutions were then mixed with C-CsF carbon powder (0.1 g.L^−1^) and were agitated at 175 rpm in an orbital incubator shaker (New Brunswick Innova^®^ 44, Paris, France) for 24 h at 298 K. After this time, the solutions were collected in amber glass chromatography vials using the same method as for the kinetic studies and were stored at −4 °C prior to analysis. The pH of all the solutions tested for kinetic and isotherm studies was the same as that of the spring water used, i.e., 7.3.

The residual concentrations of the targeted ECs were measured using a PerkinElmer Altus^®^ A-30 UPLC^®^ system coupled with a PerkinElmer QSight™ 210 triple quadrupole mass spectrometer (Perkin Elmer, Norwalk, CT, USA). A Quasar C18 (100 × 2.1 mm) column was used for the Liquid Chromatography (LC), with a mobile phase composed of mixtures of osmosed water, formic acid (>99.9%, Merck) and methanol (>99.9%, Merck). The mass spectrometer was equipped with an electrospray ionization source operating in positive and negative ion modes. Instrument control, data acquisition and processing were performed using the PerkinElmer Simplicity 3Q™ software, Version 3.0.2. Calibration curves were performed with Carbamazepine 13C6 and diclofenad-D4 as internal standards in the range of 5 µg.L^−1^. to 100 µg.L^−1^.

Equations (1) and (2) were used to calculate the ECs removal efficiency (%) and sorption capacity of beads (µg.g^−1^), respectively. In the equations, C_i_ and C_f_ denote the initial and final concentrations, respectively, w (g) specifies the amount of adsorbent and V (L) represents the volume of the contaminant solution used to perform the adsorption experiment.

(1) $$ECs\;removal\%=\frac{\left(C_{i}-C_{f}\right)}{C_{i}}\times 100$$

(2) $$ECs\;adsorption\;capacity\;\left(q_{max}\right)=\frac{\left(C_{i}-C_{f}\right)\times (V)}{w}$$

### 2.5. Kinetic and Isotherm Modeling

To identify the equilibrium sorption time and adsorption capacity, and identify the mechanism of adsorption, the kinetic modeling was performed, respectively, using pseudo-first-order [60], pseudo-second-order [61], Elovich [62], intra-particular diffusion in spherical adsorption [63] and intra-particular diffusion [64].

The integration of the pseudo-first-order relation gives Equation (3):

(3) $$q_{t}=q_{e}(1-e^{{-K}_{1}t})$$

where q_e_ and q_t_ are the adsorption capacities at equilibrium, and at time t, K_1_ is the pseudo-first-order rate constant (min^−1^).

According to the pseudo-second-order model, the kinetic equation is (4):

(4) $$q_{t}=\frac{q_{e}t}{\left(t-\frac{1}{\left(K_{2\;}q_{e}\right)}\right)}$$

where K_2_ is the pseudo-second-order rate constant (μg.g^−1^.min^−1^).

The simplified Elovich Equation (5) [62] can be expressed as follows:

(5) $$ln(q_{t})=1/\beta \;ln(\alpha .\beta )+1/\beta \;lnt$$

where *α* (μg.g^−1^.min^−1^) is the initial adsorption rate and *β* (g.μg^−1^) is the constant related to the outer surface and the chemisorption activation energy.

The intra-particular diffusion model Equation (6) from the Weber and Morris model [63] is as follows:

(6) $$q_{t}=K_{d1}{\;t}^{1/2}+C$$

where K_d1_ is the intra-particle diffusion rate constant (μg.g^−1^.min^−1/2^) and C is a constant.

Another approximate equation for intra-particle diffusion-controlled adsorption [64] is (7):

(7) $$q_{t}=q_{e}{\left(1-e^{{-K}_{d2\;}t}\right)}^{1/2}$$

where q_t_ is the adsorbed amount at t, q_e_ the adsorbed amount at equilibrium and K_d2_ is the rate constant.

The adsorption isotherms were modeled by using the Langmuir (5) equations.

The Langmuir Equation (5) is as follows:

(8) $$q_{e}=\;\frac{q_{max}\;K_{L}\;e^{\left(-\frac{{-∆H}_{ads}}{RT}\right)}}{\left(1+K_{L}{\;C}_{e}\right)}$$

where *C*_*e*_ (μg.L^−1^), q_e_ (μg.g^−1^), q_max_ (μg.g^−1^), K_L_ (L.μg^−1^), ΔH_ads_ (J.mol^−1^), R (J.K^−1^.mol^−1^) and T (K) are the equilibrium concentration in the solution, the equilibrium adsorption uptake, the maximum adsorption capacity, the Langmuir constant, the enthalpy of adsorption, the constant of perfect gas and the temperature, respectively.

The Freundlich Equation (6) is as follows:

(9) $$q_{e}=\;K_{F}{\;(C_{e})}^{1/n}$$

where K_F_ (L^1/n^.g^−1^.μg^1−1/n^) and n are the Freundlich constant and n is the correction factor, respectively.

The commonly used parameters of isotherm models (q_max_ (μg.g^−1^), K_L_ (L.g^−1^) and ΔH_ads_ (J.mol^−1^) for the Langmuir model, and K_F_ (L^1/n^.g^−1^.μg^1−1/n^) and n for the Freundlich model) were determined by fitting the experimental data using a non-linear least-square method with a trust-region algorithm. The frequently used statistical measurement (i.e., coefficient of determination (R^2^)) was applied on the obtained kinetic/isotherm parameters to confirm the suitability of the model(s).

## 3. Results and Discussion

### 3.1. Characterization of the Magnetic Nanoparticles

#### 3.1.1. XRD Pattern of the Nanoparticles and Carbon Beads

To distinguish the crystallinity and phase of the synthesized iron-oxide nanoparticles synthesized at 80 °C, the recorded XRD patterns were analyzed as shown in Figure S1a. The XRD pattern of magnetic nanoparticles coated or not with oleic acid layers shows the major peaks which matched well with the JCPDS card no. 19-0629 of Fe_3_O_4_ crystal with a spinel structure. The diffraction lines of some impurities of maghemite (Fe_2_O_3_) have also been identified in Figure S1a.

Figure S1b shows the XRD patterns of C-Cs, C-CsG and C-CsF/1% beads. The carbon from CS beads exhibits a broad diffraction feature at 2θ ≈ 25.5° and a broad band at 2θ ≈ 43° attributable to reflection (002) on disordered domains and 10. band typical of disordered turbostratic carbon, respectively. The CsG composite beads show a stronger and slightly sharpened signal in the same 2θ region (≈24–27°), which could indicate increased graphitic ordering upon GO incorporation. The presence of quartz and calcite impurities is found in the diffractogram of the C-CsG sample. The diffraction pattern of magnetite-doped carbon (C-CsF/1%) displays additional sharp reflections at 2θ ≈ 30.1°, 35.5°, 43.2°, 57.1° and 62.6°, indexed to the (220), (311), (400), (333) and (440) planes of spinel Fe_3_O_4_ (JCPDS 19-0629), confirming successful incorporation of magnetite nanoparticles into the carbon matrix. The presence of calcite peaks in this C-CsF/1% sample can be attributed to the washing step with non-controlled water after the carbonization treatment. Moreover, the presence of peaks of small intensity highlights the impurities of Fe_2_O_3_ in the form of hematite and maghemite in C-CsF/1%.

#### 3.1.2. SEM Images of the Nanoparticles

SEM images (Figure S2a,b) indicate that the nanoparticles synthesized without the addition of oleic acid are agglomerated and possess a heterogeneous 30–60 nm diameter size distribution, whereas the nanoparticles covered with oleic acid present a homogeneous 25 nm diameter size distribution.

### 3.2. Characterization of Synthesized Carbon Beads

#### 3.2.1. Surface Area and Porosity Characterization of Synthesized Samples by Gas Adsorption–Desorption

The N_2_ adsorption isotherm of the C-Cs-700 beads (obtained at 700 °C) is type I + IV (reduced hysteresis), indicating that the sample is mainly microporous (Figure S3). The N_2_ adsorption isotherms of the beads obtained in the range 800–1000 °C (C-Cs-800, C-Cs-900 and C-Cs-1000) are type IV, with hysteresis (B-type) indicating both the presence of micro and mesopores (Figure S3). At T > 800 °C, the decrease in the specific surface area is attributed to the collapse of the porous structure. Moreover, the proportion of mesopores is increasing together with the increase in temperature.

In order not to increase significantly the mesoporosity and to have mainly microporous beads, the temperature of pyrolysis for the preparation of carbon beads dedicated to the removal of micropollutants was set to 700 °C.

**Figure 1:**
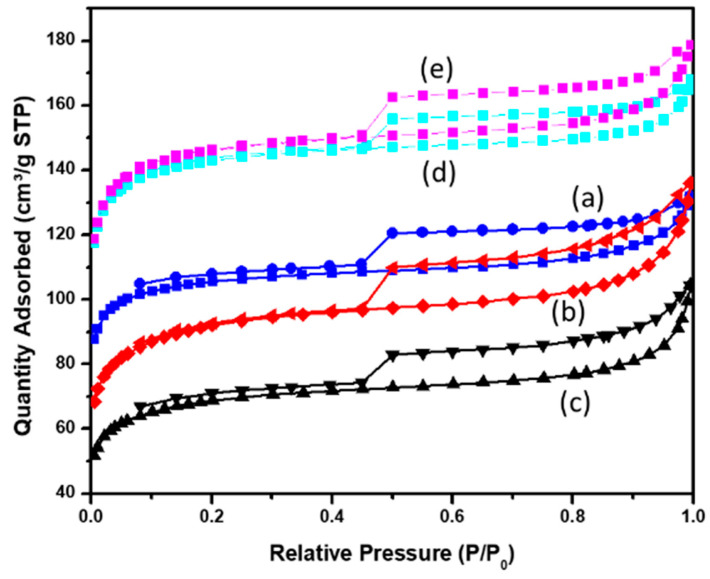
N_2_ adsorption isotherms at 77 K of AC beads pyrolyzed at 700 °C: C-CsF/1% (a), C-CsF/2% (b), C-CsF/5% (c), C-Cs-700 (d) and C-CsG (e).

Figure 1 compares the isotherms of N_2_ physisorption at 77 K of the beads prepared from pure CS and with the addition of Fe_3_O_4_ (1%, 2% and 5%) or GO. All the isotherms are Type I + IV and they differ mainly by the microporous volumes, while their mesoporous volumes are almost identical (Table 3). The inclusion of additives such as GO and Fe_3_O_4_ in the chitosan gels does not modify the mesoporous volume, suggesting that the mesopores are characteristic of the thermochemical activation process of the chitosan matrix. The pore size distributions of the samples obtained from the BJH (Barrett Joiner Halenda) model applied to N_2_ adsorption isotherms at 77 K (Figure S4), indicate that the mesoporous volumes of the beads prepared at 700 °C are almost similar. This mesoporous volume clearly increases with the heat treatment temperature.

The addition of GO with activated CS allows the porous volume to slightly increase (Table 3), but the difference in BET surface area values between C-CsG (572 m^2^.g^−1^) and C-Cs-700 (561 m^2^.g^−1^) is weak. The addition of magnetite from 1 mass. % to 5 mass. % clearly decreases the microporous volume from 0.16 cm^3^.g^−1^ to 0.1 cm^3^.g^−1^, while the mesoporous volume remains constant (~0.05 cm^3^.g^−1^). This means that the iron oxide particles in these compounds probably block the micropore entry or the connection between the micropore network. In order to maximize the micropore volume in further adsorption studies of micropollutants, we have preferred to use the sample containing the lowest amount of iron nanoparticles (i.e., C-CF/1%) and possessing the highest BET specific surface area and micropore volume.

The pore size distributions of the samples obtained from the BJH (Barrett Joiner Halenda) model applied to N_2_ adsorption isotherms at 77 K (Figure S4) indicate that the mesoporous volumes of the beads prepared at 700 °C are almost similar. This mesoporous volume clearly increases with the heat treatment temperature.

#### 3.2.2. Morphological and Compositional Analysis of Samples: SEM-EDS and TEM

**Figure 2:**
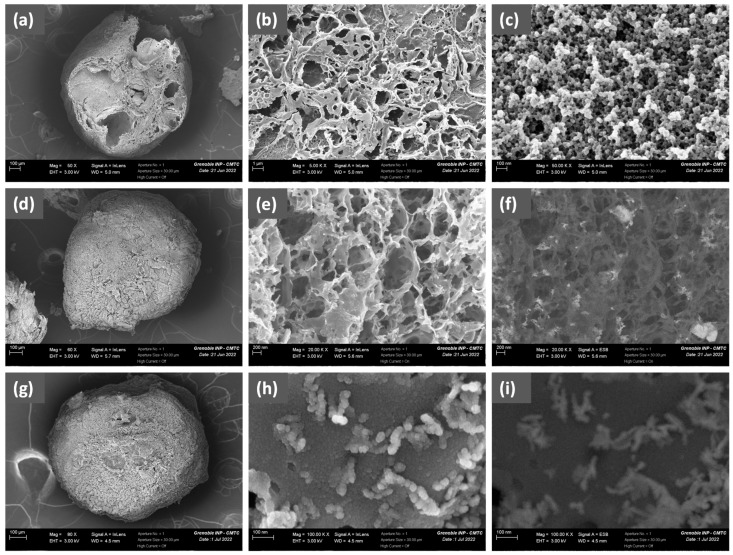
SEM images of C-Cs-700: (**a**) whole bead, (**b**) zoom of surface of the bead (magnification ×5 k) and (**c**) magnified image (×50 k) of the carbon nanoparticles arrangement on the surface. SEM images of C-CsF/2%: (**d**) whole bead, (**e**) zoom of the bead surface (×20 k) and (**f**) previous image obtained in the backscattered electron mode. SEM image of C-CsF/1%: (**g**) whole bead, (**h**) zoom of the bead surface showing iron oxide nanoparticles (×100 k) and (**i**) previous image obtained in the backscattered electron mode.

The SEM image of the C-Cs-700 sample (Figure 2a) shows that the shape of the beads is preserved after pyrolysis but contains few large craters formed by the emitted gas during pyrolysis. The texture of the bead surface (Figure 2b) is formed by the presence of micrometric macropores (0.5–5 µm), some of which are crater shaped. The walls of the macropores can be composed of an arrangement of agglomerated carbon nanoparticles with an average diameter of 20–30 nm (Figure 2c), and mesopores are observed in between these unit particles.

Figure 2d,g shows examples of C-CsF/1% and C-CsF/2% beads, which are less porous than the C-Cs-700 ones. Small macropores with submicrometric size (0.1–0.8 µm) are observed in the image of C-CsF/2% beads (Figure 2e). The presence of iron nanoparticles of various sizes in the C-CsF/2% beads is evidenced by the image obtained in back-scattered electron mode (Figure 2f), as well as by EDS microanalysis reporting 7.7 mass. % iron in the global surface analysis. The SEM image in secondary electron mode shows that the surface of the C-CsF/1% beads is decorated with agglomerates of spherical elemental nanoparticles with beads of a 25–30 nm diameter (Figure 2h). The same image obtained in back-scattered electron mode (Figure 2i) demonstrates that the clearer domains are iron oxide particles. EDS microanalysis reported 3.3 mass. % iron in the global analysis of the C-CsF/1% sample surface.

**Figure 3:**
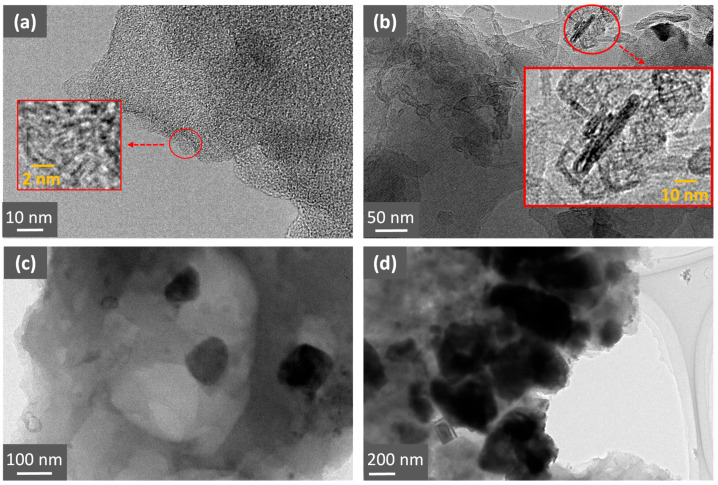
TEM images of (**a**) C-Cs-700 sample (obtained at 700 °C), (**b**) C-CsG sample, (**c**) C-CsF/1% (1 mass. % Fe_3_O_4_) and (**d**) C-CsF/2% (2 mass. % Fe_3_O_4_).

TEM images of all the samples are shown in Figure 3. The C-Cs-700 TEM image (Figure 3a) shows the typical microstructure of disordered AC, consisting of a more or less random arrangement of units of fewer than a few tens of flat or pleated, stacked graphene layers, each of which extends around 2 nm along the *ab* planes (L*_a_*) (see enlarged insert area in Figure 3a). Indeed, the nanostructure of carbon C-Cs-700, as observed by TEM, can be interpreted as a ‘crumpled paper’ at a microscopic scale, which is arguably the most accurate description of this nanostructure. TEM does not allow micropores in the carbon matrix to be observed, but it is thought that they are in the spaces between neighboring graphene layers. Mesopores were not observed in the TEM images of the surface zones of C-Cs-700.

The TEM image of the C-CsG sample (Figure 3b) shows the presence of multilayer graphene sheets (see enlarged insert area in Figure 3b), either flat or curved, with limited L_c_ (L_c_ < 10 nm) and high dimensions along the *a* axis (L_a_: coherence domain size, 20 nm < L_a_ < 1 μm), compared to the C-Cs-700 nanostructure. This confirms the presence of two types of carbons within C-CsG beads: disordered carbon from chitosan pyrolysis and graphene oxide (GO) heat-treated carbon.

Iron oxide nanoparticles in the carbon matrix are visible in the TEM image of C-CsF/1% (Figure 3c), with diameters varying from 25 to 100 nm (initial Fe_3_O_4_ nanoparticle diameter 25 nm). This indicates that heat treatment leads to agglomeration, coalescence and growth of the particles, resulting in an increase in the size of the pristine iron oxide particles. In the C-CsF/2% sample, particles with a larger diameter (50 nm to 1 µm) are observed (Figure 3d), indicating that the higher the Fe_3_O_4_ content, the larger the particles formed during pyrolysis from the pristine nanoparticles.

#### 3.2.3. Chemical Characterization of Samples by XPS

**Figure 4:**
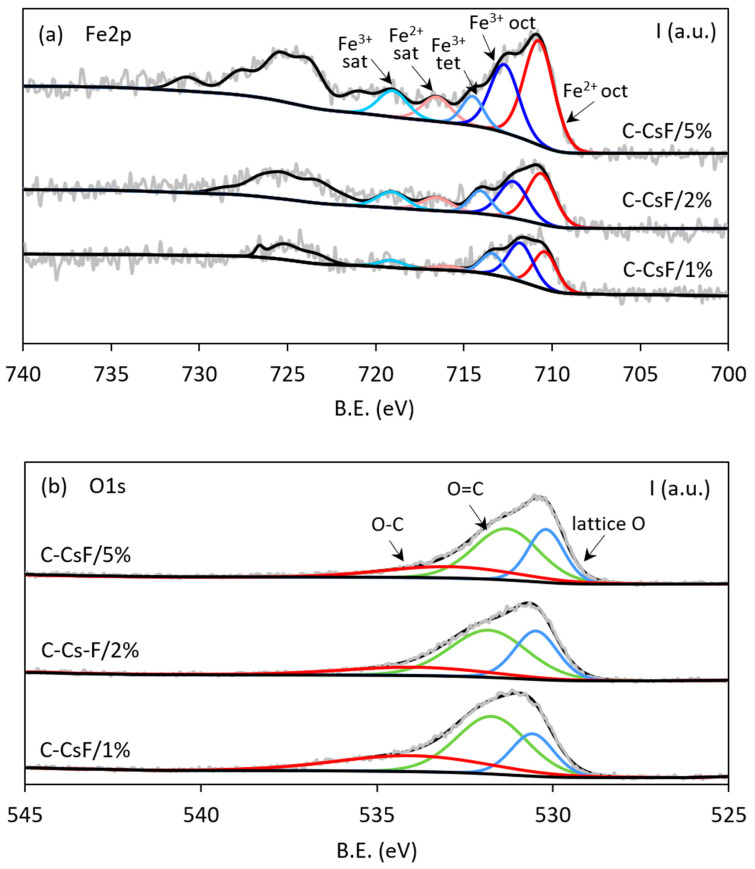
Fe2p (**a**) and O1s (**b**) XPS spectra of samples C-CsF/1% (1 mass. % Fe_3_O_4_), C-CsF/2% (2 mass. % Fe_3_O_4_) and C-CsF/5% (5 mass. % Fe_3_O_4_).

The Fe 2p XPS spectra of the three magnetite-containing samples (Figure 4a) show the presence of two broad ranges of peaks corresponding to the Fe 2p3/2 (from ~721.4 to 708.2 eV) and Fe 2p1/2 (from ~732.8 to 719.3 eV) signals [65]. The fitting of the Fe 2p3/2 region revealed five peaks: the three peaks of the highest intensity can be attributed to different oxidation states and environments, whereas the two lower-intensity peaks can be attributed to satellite peaks [66]. XPS spectra clearly evidence the presence of two oxidation states, Fe^2+^ and Fe^3+^. The lowest binding energy peak in the 713.7–708.2 eV range is attributed to Fe^2+^, with a corresponding satellite in the 718.6–714.6 eV range. Whereas the Fe^2+^ signal is in the characteristic energy range of octahedrally coordinated Fe^2+^ [67], the Fe^3+^ ions are distributed over both octahedral (peak in the 715.4–709.9 eV range) and tetrahedral (peak in the 716.1–713.1 eV range) sites, with a corresponding satellite in the 721.4–717.1 eV range. The fitting of the 2p1/2 pattern could also be performed with the five characteristic peaks described above, but given their lower intensity, peak positions are debatable, and their intensities are therefore less accurate. However, after considering the three Fe^2+^ and Fe^3+^ characteristic peaks of each pattern, the 2p3/2 to 2p1/2 ratio was found to be ~2.3 for the three samples, which agrees with the theoretical value of 2. The total iron content in the samples follows a logical order in relation to the amounts of iron salt introduced into the gel, even if the ratio is not respected (Table 4). Samples C-CsF/2% and C-CsF/5% contain, respectively, about 1.4 and 2.6 times that of the sample C-CsF/1%. The Fe^3+^/Fe^2+^ ratio of the three samples ranges between 0.83 (for the sample prepared with the highest amount of iron salts) and 1.65 (for the one prepared with the lowest amount of iron salts), which is lower than the expected value of 2 for stoichiometric Fe_3_O_4_. These variations indicate that samples not only contain magnetite but also other iron oxides such as FeO, for which the amount is the highest in sample C-CsF/5% Fe_3_O_4_. This hypothesis is supported by the increase in relative intensity of the Fe 2p3/2 peak attributed to Fe^2+^ in the 713.7–708.2 eV range as the amount of iron salts introduced increased (Figure 4).

The O/Fe ratio of 1.34 expected for magnetite was obtained for sample C-CsF/1% and almost for sample C-CsF/2%. However, this ratio is much lower for sample C-CsF/5% (Table 4: O/Fe = 0.58), suggesting an enrichment in iron relative to oxygen and thus confirming the probable presence of FeO.

The O1s XPS spectra of the synthesized samples (Figure 4b) can be fitted with three peaks. According to the literature, the lowest energy peak (in the 531.9–528.9 eV range) can be assigned to the oxygen in the Fe_3_O_4_ lattice [67]. The peaks in the 534.5–528.8 eV and 538.1–528.7 eV regions can be assigned to O=C and O-C bonds, respectively. An increase in the relative intensity of the peak assigned to Fe_3_O_4_ is observed (Figure 4b) as the iron salt content in the reaction mixture increases. The intensity of the signal assigned to Fe_3_O_4_ corresponds to ~20% of the total O signal for the sample prepared with the lowest salt content (sample C-CsF/1%), whereas it corresponds to ~28% for the other two samples. Surprisingly, the intensity of this signal seems too low for the sample containing the highest amount of iron (C-CsF/5%) compared to the other two samples. However, the signal of the suspected FeO is in the same energy region and fitting is thus not as accurate.

### 3.3. Adsorption of ECs onto Carbon Adsorbents

#### 3.3.1. Kinetics

The kinetics of the adsorption of the whole beads of C-Cs-700, C-CsF/1% and C-CsG were first tested and compared under the conditions described in Section 2.4 (Figure S5). For all the ECs, the adsorption kinetics are very slow and equilibrium has not yet been reached with C-CsF/1% and C-CsG, as the concentration of the adsorbed species is still increasing after 24 h. A plateau of equilibrium can be observed for all the ECs after 3 h of adsorption on C-CsF/1%. It was observed for all sample types that, due to agitation, the beads disaggregated into smaller particles owing to their relatively weak mechanical strength. The mechanical strength of the beads follows the trend C-CsF/1% > C-Cs-700 > C-CsG. For application, C-CsF/1% is the best sample, which can be removed magnetically from the solution (Figure S6) and which can adsorb quickly. However, to achieve complete removal of the micropollutants at an initial concentration of 10 µg.L^−1^, a quantity of C-CsF/1% bead adsorbent around 1 g.L^−1^ would be required.

**Figure 5:**
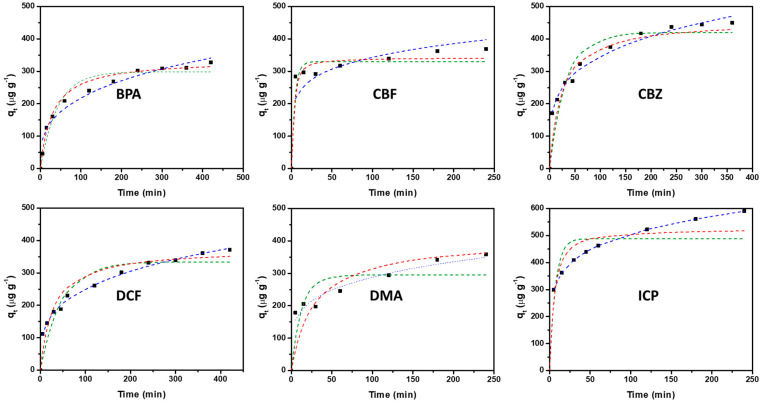
Kinetics of adsorption of the ECS (BPA, CBF, CBZ, DCF, DMA and ICP) of C-CsF/1% (1 mass. % Fe_3_O_4_) carbon micrometric powder: experimental data (black square) and dotted lines representing the models of pseudo first order (green), of pseudo second order (red) and of Elovich (blue).

In order to accelerate the kinetics of the adsorption of the C-CsF/1% sample, which are controlled by diffusion inside the beads, and to increase the adsorption capacities, the powder of this sample was used as an adsorbent. The kinetics of ECs adsorption on C-CsF/1% powder were studied under the conditions described in Section 2.4. The results of the kinetics of adsorption are presented in Figure 5. For all the contaminants, the adsorption quantity is increasing with the contact time. The values of maximum adsorption are in the same order of magnitude (350–600 μg.g^−1^) and the plateau of equilibrium is still not attained at t = 250 min. This suggests that, at the studied initial concentration (i.e., 50 μg.L^−1^), an absence of competition for adsorption occurs between the contaminants in the mixture, and that enough sites of adsorption are present so that all the contaminants can be adsorbed on these sites. Thus, the application of models of kinetics (or isotherms in the same order of magnitude of concentration) involving only a single contaminant can be applied.

In a first modeling study, the adsorption kinetics of the studied contaminants were evaluated using the non-linear forms of the pseudo-first-order and pseudo-second-order kinetic models, and the Elovich models. The obtained rate parameters with the correlation coefficients are presented in Table 5. For the pseudo-first-order model, the correlation coefficients (R^2^) ranged from 0.780 (DMA) to 0.958 (BPA), indicating that this model is less relevant than the pseudo-second-order model, for which the values of R^2^ are higher than 0.794. The best agreement between the experimental and calculated adsorption capacities (q_e_) was found using the Elovich model (R^2^ values are higher than 0.977). Indeed, Figure 5 shows that this model fits well with the experimental data, even though the adsorption mechanism is clearly not chemisorption.

**Figure 6:**
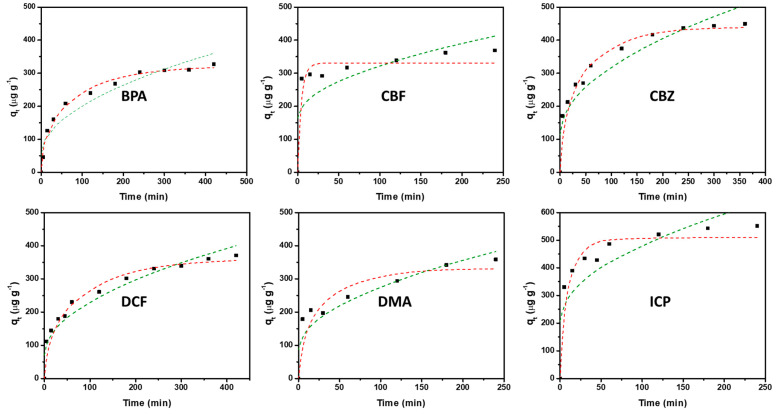
Kinetics of adsorption of the ECs (BPA, CBF, CBZ, DCF, DMA and ICP) of C-CsF/1% (1 mass. % Fe_3_O_4_) carbon powder: experimental data (black square) and dotted lines representing the models of intra-particular diffusion of Weber and Morris (green) and of intra-particle diffusion-controlled adsorption of Vermeulen (red).

Secondly, the adsorption kinetics of the studied contaminants were evaluated using diffusion models such as the intra-particular diffusion model of Weber and Morris and the intra-particle diffusion-controlled adsorption model of Vermeulen (Figure 6) for which the rate parameters together with the correlation coefficients are presented in Table 6. The comparison of the correlation coefficients (R^2^) of these two models indicates that the model of diffusion-controlled adsorption of Vermeulen adequately fitted the adsorption kinetics for most of the studied ECs (R^2^ values ranged from 0.887 (DMA) to 0.989 (BPA)). The good agreement between the experimental and calculated adsorption capacities (q_e_) further supports the good applicability of this model and indicates that the adsorption kinetics of the ECs is clearly controlled by intra-particle diffusion phenomenon.

**Figure 7:**
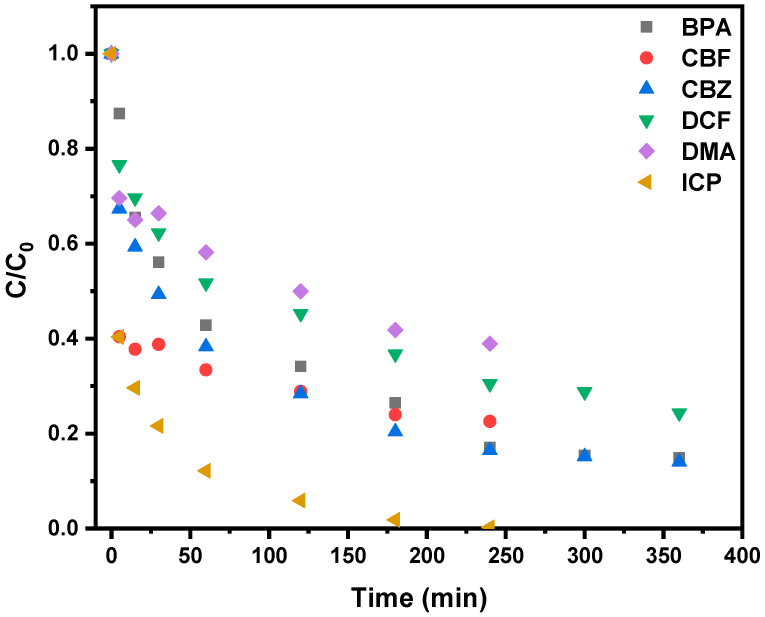
Kinetics of adsorption of the ECs (BPA, CBF, CBZ, DCF, DMA and ICP) of C-Cs-F (1 mass. % Fe_3_O_4_) carbon micrometric powder.

The comparison of the kinetics representing the evolution with time of the C/C_0_ ratio is shown in Figure 7. It shows that the speed of adsorption follows the trends ICP > CBZ > CBF > BPA > DCF > DMA. Moreover, the calculated rate constants of the second-order model (K_2_) exhibited wide variations across the different ECs (Table 5), attributable to physicochemical properties of the contaminants. ECs with significant polarizability, which are among the more hydrophilic (with low K_ow_ values), containing aromatic cycles, polar substituents and electron-donating groups such as ICP and CBF, exhibited relatively higher K_2_ values. This suggests enhanced interactions, possibly through H-bonding and π–π electron donor–acceptor interactions. By contrast, DMA and DCF molecules showed comparatively lower speed of adsorption. This is likely due to their pKa values, which are around 4.5, indicating that these molecules are negatively charged at the pH of the natural water (7.3), leading to electrostatic repulsion with the adsorbent surface which limits surface affinity and diffusion into the pores. Moreover, the DMA molecule is an aliphatic one possessing the lowest polarizability value among the studied ECs, and thus, its attraction through London forces was limited by contrast with other molecules.

#### 3.3.2. MECs Adsorption Isotherms

**Figure 8:**
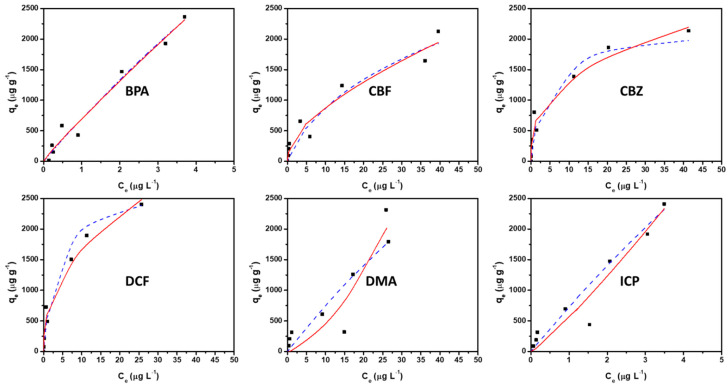
Adsorption isotherms of the ECs (BPA, CBF, CBZ, DCF, DMA and ICP) of C-CsF/1% (1 mass. % Fe_3_O_4_) carbon powder: experimental data (black square), dotted blue lines representing the fitted model of Langmuir according to Equation (5) and red line representing the fitted model of Freundlich according to Equation (6).

The profile of the isotherms of adsorption (Figure 8) is typically linear for ICP, BPA and DMA and can be described either by Langmuir type isotherms (Equation (5)) with a constant K_L_ value close to zero (Table 7) or with a Freundlich simulation (Equation (6)) with a constant K_F_ value close to one (Table 8). These typical linear isotherms for ICP, BPA and DMA indicate that Henry’s law applies to what is known in this range of very low concentration. Indeed, the refined values of the Freundlich correction factor (n) are close to unity for ICP, BPA and DMA (Table 8), confirming the applicability of Henry’s law.

A comparison of the R^2^ coefficients of determination in Table 7 and Table 8 indicates that the Freundlich model better reproduces the experimental isotherms of the ECs than the Langmuir model, except for ICP and DMA. The appropriate model to describe the adsorption of all the contaminants can be either the model of Freundlich or the model of Langmuir (Table 7 and Figure 8). However, the Freundlich model is only an empirical model and is not based on adsorption theory.

A non-linear profile of isotherms for CBZ, CBZ and DCF was identified, indicating a propensity for a decline in the adsorption capacity gain with increasing contaminant concentrations. (Figure 8). CBZ, CBZ and DCF molecules are among the least soluble ones and the most hydrophobic, with high values of Log(K_ow_). The AC C-CsF/1% is assumed to have some hydrophilic sites with the presence of oxygenated surface groups. Thus, the limitations of the adsorption of CBZ, CBZ and DCF at concentrations higher than 20–25 µg.L^−1^ could be related to their hydrophobic character and the limited amount of hydrophobic adsorption sites on the AC sample, which can explain the Langmuir-type profile of the isotherms. Moreover, the DCF is negatively charged at the pH of the natural water. The isoelectric pH of C-CsF was measured at pH 4 by zetametry. Electrostatic repulsions are thus supposed to occur between this molecule and the negatively charged surface of the adsorbent probed by zeta potential measurements (probably due to the presence of oxygenated acidic groups at the surface of the carbon adsorbent), which can limit adsorption at concentrations higher than 20 µg.L^−1^.

The strength of adsorption interactions of the ECs at very low concentration (lower than 5 µg.L^−1^) can be estimated from the values of the Henry constant reported in Table 7, which shows the following trend: ICP > DCF > BPA > CBF > CBZ > DMA. This confirms that the IMP molecule has a higher affinity for C-CsF/1%, while DMA has the lowest affinity, in agreement with the results of kinetics. Surprisingly, DCF also has a high affinity for C-CsF/1%. This could be related to its low solubility (lowest solubility value) and hydrophobic character (lowest value of log(K_ow_)), which promote the adsorption at low concentration on a limited number of hydrophobic aromatic sites of the AC. BPA and CBZ, which also possess a limited solubility, show a high value of Henry’s constant attributed to the adsorption on hydrophobic sites. The values of adsorption enthalpies were estimated from the fit with the Langmuir model (Table 7). They all indicate that the ECs are rather physiosorbed on the magnetic AC C-CsF/1%.

## 4. Conclusions

We prepared AC beads using thermal activation of chitosan hydrogel beads. The beads were mainly microporous while prepared at 700 °C. They were doped with magnetite (Fe_3_O_4_) nanoparticles or graphene oxide. The AC beads doped with 1 mass. % Fe_3_O_4_ were characterized by SEM, TEM, XPS and N_2_ adsorption–desorption measurements at 77 K, revealing the presence of nanoparticles of Fe_3_O_4_ in the porous carbon.

A commercial spring water contaminated with a mixture of micropollutants (bisphenol A, carbofuran, carbamazepine, diclofenac, dimethoate and imidacloprid) at a concentration level of 50 μg.L^−1^ for each contaminant (0.1 g.L^−1^ of adsorbent), was purified with the powder resulting from the bead grinding. The adsorption rate is 50 to 99% of the initial amount after 4 h of contact time. The kinetics of adsorption is controlled by intraparticle diffusion.

The adsorption isotherms of the mixture of pollutants on AC powder doped with 1 mass. % Fe_3_O_4_ has demonstrated the possibility to adsorb at least 95% of a mixture of the micropollutants with a concentration of 50 μg.L^−1^ each. The isotherms of adsorption were found to be either of the Langmuir or Henry type. The adsorption was assumed to be mainly governed by physisorption, probably through hydrophobic interactions in micropores at very low concentrations (<5 ppb), while at higher concentrations (>20 ppm), hydrophilic interactions due to surface functional groups (O and N surface groups) might be dominant.

Thus, the potential for application of these materials is significant in the domain of purification of drinking water, especially since it is possible to separate them magnetically from the liquid medium, which is a definite advantage.

## Figures and Tables

**Table 1:** Formulas and phyco-chemical properties of the studied contaminants.

Molecule Name	Bisphenol A (BPA)	Carbofuran (CBF)	Carbamazepine (CBZ)	Diclofenac (DCF)	Dimethoate (DMA)	Imidacloprid (ICP)
Molecular formula	C_15_H_16_O_2_	C_12_H_15_NO_3_	C_15_H_12_N_2_O	C_14_H_11_Cl_2_NO_2_	C_5_H_12_NO_3_PS_2_	C_9_H_10_ClN_5_O_2_
Structure	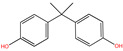	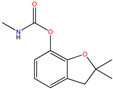	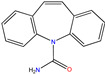	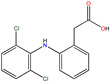	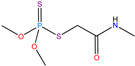	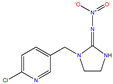
Polarizability (Å^3^)	25.4	23.3	25	27.9	21.4	23.2
Water solubility (mg.L^−1^)	120–300	351	17.7	2.37	39,000	510
Log K_ow_	3.64	2.32	2.45	4.51	0.70	0.57
pKa	9.6	11.95	13.90	4.15	4.20	1.56/11.12
Usage	Polycarbonate precursor	N-methyl carbamate insecticide and nematicide	Analgesic, anti-epileptic	Analgesic, anti-inflammatory	Organophosphorus insecticide	Neonicotinoid insecticide

**Table 2:** Conditions of preparation and composition of various AC beads derived from NaOH impregnated CS pyrolysis.

Sample Name	CS Concentration in Acetic Acid Solution (Mass. %)	IR of NaOH (%) *	Gelification Time (h)	Fe_3_O_4_ Addition (Mass. % of Solid)	GO Addition (Mass. %)	Pyrolysis T (°C)
C-Cs-T ^#^	5	~5–20	1–6	-	-	600–1000
C-CsF/1%	5	~9	2	1	-	700
C-CsF/2%	5	~9	2	2	-	700
C-CsF/5%	5	~9	2	5	-	700
C-CsG	2.5	~9	2	-	~8	700

* The impregnation ratio (IR) is defined as the percentage of NaOH in the beads relative to the mass of CS. ^#^ T is temperature of pyrolysis expressed in °C.

**Table 3:** Textural properties of the AC beads pyrolyzed in the range 700–1000 °C: C-CsF/1%, C-CsF/2%, C-CsF/5%, C-Cs-700, C-Cs-800, C-Cs-900, C-Cs-1000 and C-CsG.

AC Beads Type	S_BET_ ^§a^ (m^2^.g^−1^)	V_micro_^§b^ (cm^3^.g^−1^)	V_meso_^§c^ (cm^3^.g^−1^)	V_total_^§d^ (cm^3^.g^−1^)
C-Cs-1000	666	0.24	0.73	0.97
C-Cs-900	684	0.26	0.45	0.71
C-Cs-800	776	0.33	0.20	0.53
C-Cs-700	561	0.22	0.04	0.26
C-CsF/1%	415	0.16	0.04	0.20
C-CsF/2%	346	0.14	0.07	0.21
C-CsF/5%	260	0.10	0.06	0.16
C-CsG	572	0.23	0.05	0.28

^§^: from N_2_ at 77 K; ^a^: P/P_0_ = 0.01–0.05; ^b^: ∅_pores_ < 2 nm; ^c^: 2 nm < ∅_pores_ < 50 nm; ^d^: determined at P/P_0_ = 0.995.

**Table 4:** Amount of iron and oxygen elements in different forms obtained from the XPS Fe2p and O1s signals of C-CsF/1%, C-CsF/2% and C-CsF/5% samples (with various content of Fe_3_O_4_: 1 mass. %., 2 mass. % and 5 mass. %).

AC Name	Fe Total (at. %)	Fe^3+^/Fe^2+^	Fe^3+^ Oct.	Fe^3+^ Tet.	Fe^3+^ Oct./ Fe^3+^ Tet.	O Lattice	O/Fe
C-CsF/1%	5.93	1.65	2.51	1.18	2.13	7.97	1.34
C-CsF/2%	8.20	1.10	3.52	1.72	2.04	9.99	1.22
C-CsF/5%	15.56	0.83	5.34	1.73	3.08	9.01	0.58

**Table 5:** Kinetic parameters for different models applied to ECs mixture adsorption on C-CsF/1 mass% powder (Ci = 50 µg.L^−1^).

EC Name	q_e(exp)_ (µg.g^−1^)	Pseudo-First-Order Rate Parameters			Pseudo-Second-Order Rate Parameters			Elovitch Parameters		
		q_e(cal)_ (µg.g^−1^)	K_1_ (min^−1^)	R^2^	q_e(cal)_ (µg.g^−1^)	K_2_ (g.µg^−1^.min^−1^)	R^2^	α (g.µg^−1^.min^−1^)	β (µg.g^−1^)	R^2^
BPA	327	298	0.024	0.958	336	1 × 10^−4^	0.987	1.5 × 10^5^	3.28	0.977
CBF	369	330	0.386	0.946	342	2.1 × 10^−3^	0.959	1.7 × 10^13^	6.32	0.921
CBZ	449	419	0.033	0.891	455	1 × 10^−4^	0.948	1.7 × 10^8^	4.27	0.992
DCF	371	334	0.022	0.897	373	1 × 10^−4^	0.946	1.0 × 10^6^	3.57	0.996
DMA	359	295	0.091	0.780	400	1 × 10^−4^	0.794	1.2 × 10^9^	4.77	0.977
ICP	552	487	0.186	0.912	525	5 × 10^−4^	0.964	4.3 × 10^12^	5.69	0.979

**Table 6:** Kinetic parameters for the diffusion models applied to the ECs mixture adsorption on C-CsF/1% powder (Ci = 50 µg.L^−1^).

EC Name	q_e(exp)_ (µg.g^−1^)	Parameters of Diffusion Model of Weber and Morris			Parameters of Diffusion-Controlled Adsorption of Vermeulen		
		Kd_1_ (µg.g^−1^. min^−1/2^)	C (µg.g^−1^)	R^2^	Kd_2_ (min^−1^)	q_e(cal)_ (µg.g^−1^)	R^2^
BPA	327	15.22	48.4	0.924	0.0081	322	0.989
CBF	369	16.07	163.5	0.557	0.2382	331	0.948
CBZ	449	20.73	113.1	0.882	0.0139	440	0.972
DCF	371	16.08	70.4	0.940	0.0082	361	0.975
DMA	359	19.16	85.8	0.860	0.022	331	0.887
ICP	552	27.46	208.3	0.691	0.064	509	0.942

**Table 7:** Parameters for Langmuir models applied to ECs mixture adsorption isotherms on C-CsF powder (C_i_ in the range 5–250 µg.L^−1^).

	q_max_ (µg.g^−1^)	K_L_ (L.µg^−1^)	ΔH_ads_ (kJ.mol^−1^)	R^2^	K_H_ (µg.g^−1^.mol^−1^)
BPA	10,009	0.051	−0.9	0.9776	739
CBF	2700	0.049	−0.2	0.9266	142
CBZ	2010	0.301	−0.2	0.9076	642
DCF	2467	0.310	−0.2	0.9493	763
DMA	1900	0.008	−4.2	0.9604	71
ICP	10,000	0.047	−1.2	0.9603	767

**Table 8:** Parameters for Freundlich models applied to ECs mixture adsorption isotherms on C-CsF powder (C_i_ in the range 5–250 µg.L^−1^).

	K_F_ (L^1/n^.g^−1^.μg^1−1/n^)	n	R^2^
BPA	706	1.1	0.9780
CBF	269	1.86	0.9542
CBZ	633	2.99	0.9699
DCF	655	2.44	0.9817
DMA	9.91	1.63	0.8766
ICP	544	0.86	0.9362

## Data Availability

The data will be provided upon request.

## Supplementary Materials

The following supporting information can be downloaded at https://www.mdpi.com/article/10.3390/molecules30224443/s1, Figure S1: XRD patterns of the synthesized bare and coated iron-oxide nanoparticles (a) and carbon beads (b). The Miller indexes of the diffraction peaks of Magnetite (Fe_3_O_4_), Calcite (CaCO_3_), Hausmanite (Mn_3_O_4_) and Quartz (SiO_2_) are labelled in blue, green, red and black, respectively. In Figure S1a, the presence of impurities of Maghemite (Fe_2_O_3_) is labelled by *. In Figure S1b, the presence of impurities of Hematite (Fe_2_O_3_), Maghemite, and Calcite is labelled by #, * and §; Figure S2: SEM microphotographs of synthesized bare (a) and oleic acid coated (b) iron-oxide nanoparticles; Figure S3: N_2_ adsorption isotherms at 77 K of C-Cs-T beads pyrolyzed at various temperature (T=700 °C, 800 °C, 900 °C and 1000 °C). The BET specific surface areas are also indicated; Figure S4: Pore size distributions obtained from the BJH (Barrett Joiner Halenda) model applied to N_2_ adsorption isotherms at 77 K; Figure S5: Kinetics of adsorption of the ECS (BPA, CBF, CBZ, DCF, DMA and ICP) of beads of C-Cs-700 (black square), C-CsG (red circle), and C-CsF/1% (1 mass. % Fe_3_O_4_) (blue triangle). The lines are guides for the eyes. Figure S6: Illustration of the magnetic properties of C-CsF/1% (1 mass. % Fe_3_O_4_) and C-CsF/5% (5 mass. % Fe_3_O_4_).
