# Supplementary material for: Magnetite-Doped Activated Carbon Beads and Powder Derived from Chitosan for Adsorption of Emerging Contaminants in Drinkable Water
Source: Molecules. 2025 Nov 18;30(22):4443. doi: 10.3390/molecules30224443 (PMC12655546; doi:10.3390/molecules30224443)
Supplement: Supplementary file 1 [file molecules-30-04443-s001.zip › molecules-3937050-supplementary.pdf]

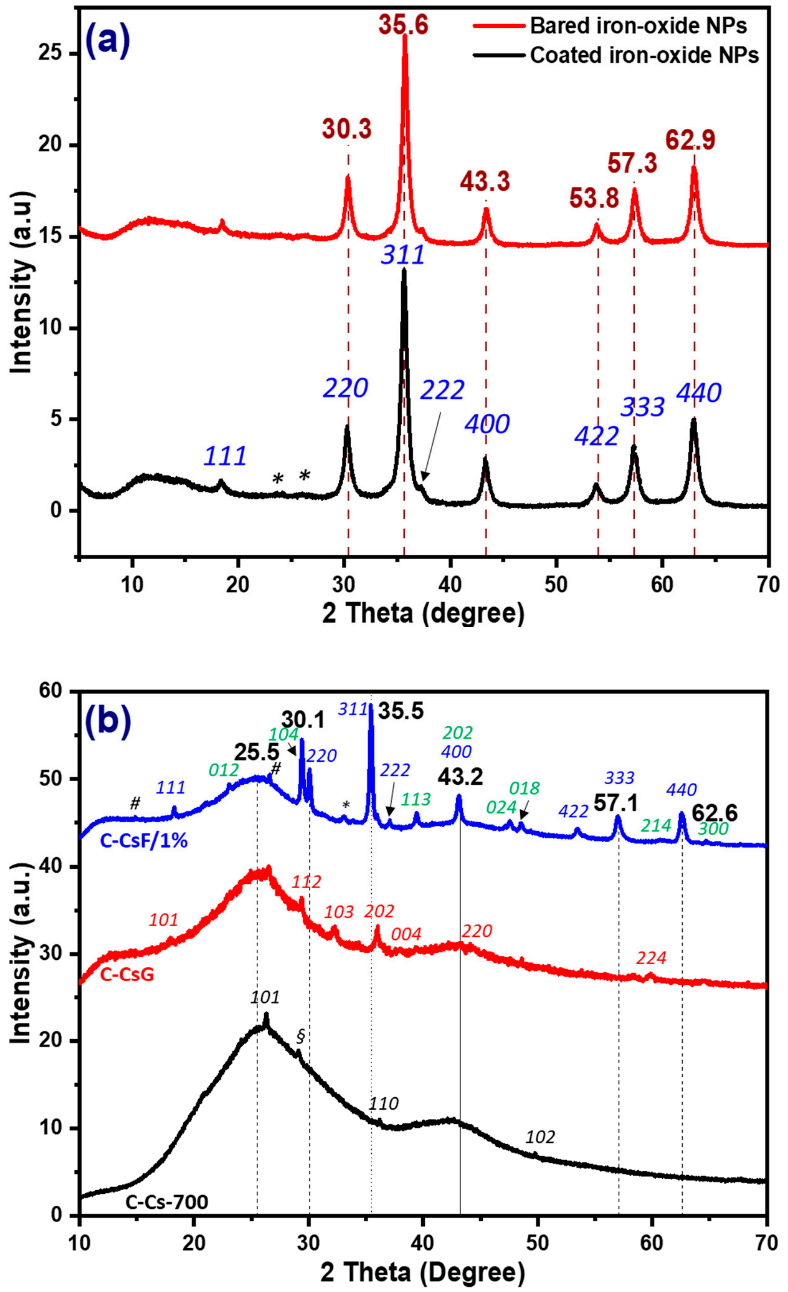

Fig. S1. XRD patterns of the synthesized bare and coated iron-oxide nanoparticles (a) and carbon beads (b). The Miller indexes of the diffraction peaks of Magnetite (Fe_3O_4), Calcite ($CaCO_3$), Hausmanite (Mn_3O_4) and Quartz (SiO_2) are labelled in blue, green, red and black, respectively. In Fig. S1(a), the presence of impurities of Maghemite (Fe_2O_3) is labelled by *. In Fig. S1(b), the presence of impurities of Hematite (Fe_2O_3), Maghemite, and Calcite is labelled by #, * and §.

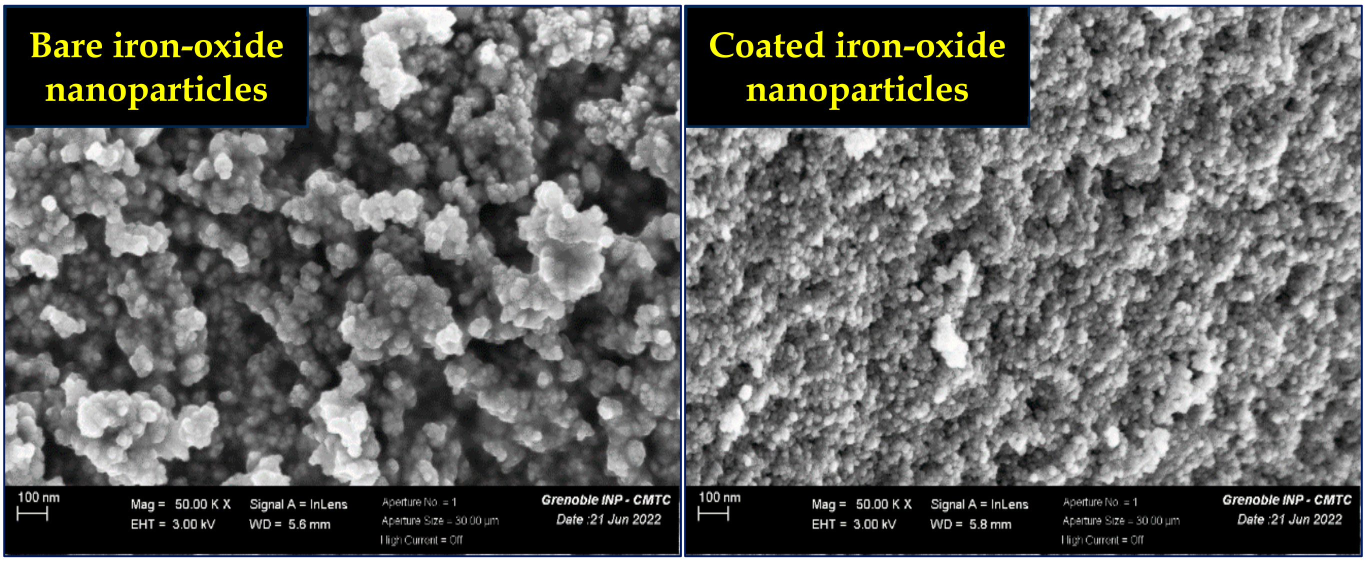

Fig. S2. SEM microphotographs of synthesized bare (a) and oleic acid coated (b) iron-oxide nanoparticles.

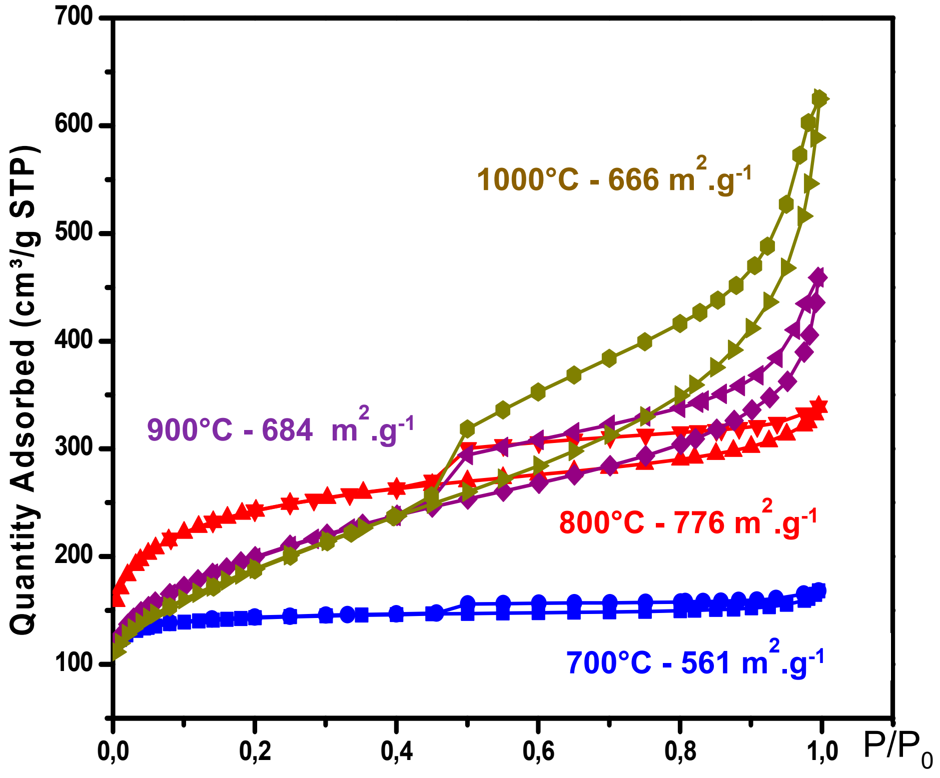

Fig. S3. N_2 adsorption isotherms at 77 K of C-Cs-T beads pyrolyzed at various temperature (T=700°C, 800°C, 900°C and 1000°C). The BET specific surface areas are also indicated.

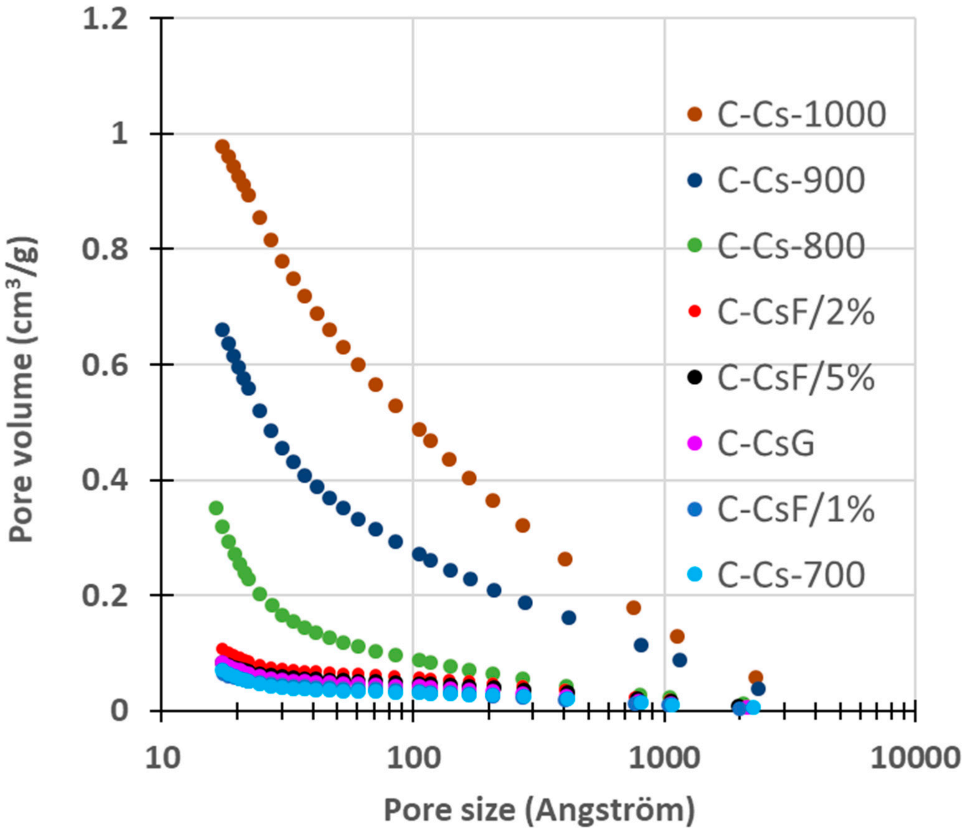

Fig. S4. Pore size distributions obtained from the BJH (Barrett Joiner Halenda) model applied to N_2 adsorption isotherms at 77 K.

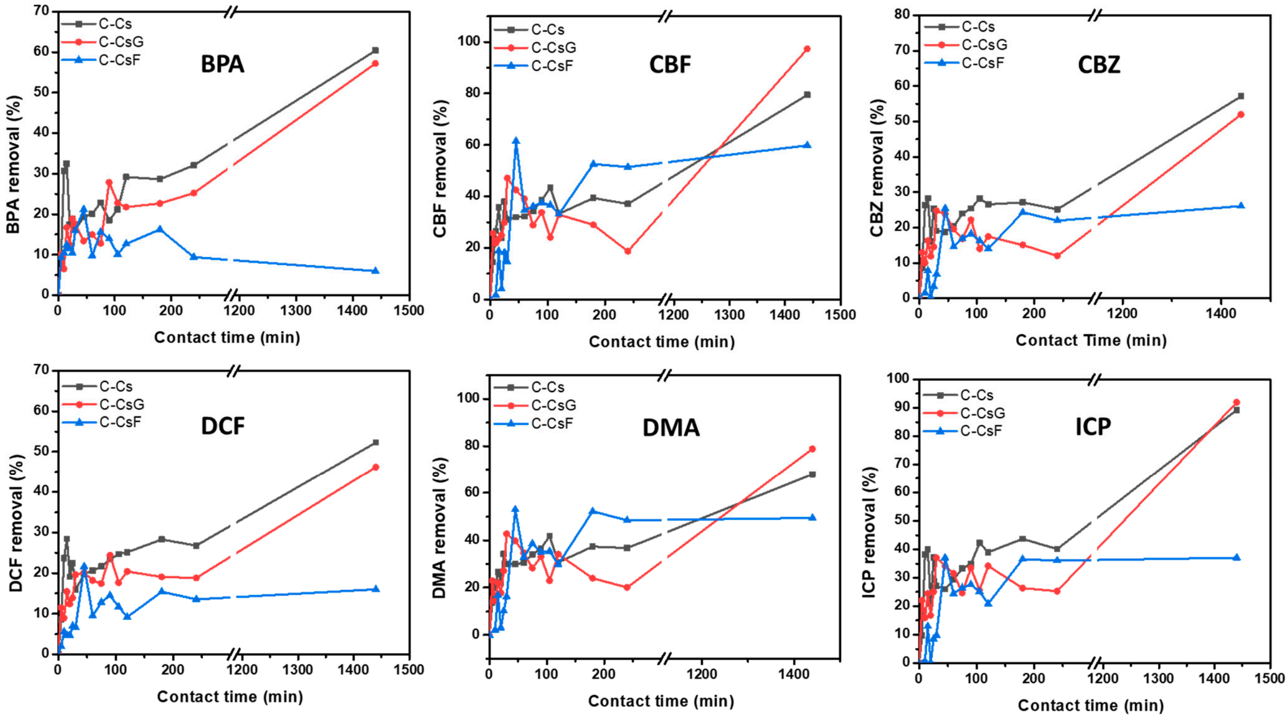

Fig. S5. Kinetics of adsorption of the ECS (BPA, CBF, CBZ, DCF, DMA and ICP) of beads of C-Cs-700 (black square), C-CsG (red circle), and C-CsF/1% (1 mass. % Fe_3O_4) (blue triangle). The lines are guides for the eyes.

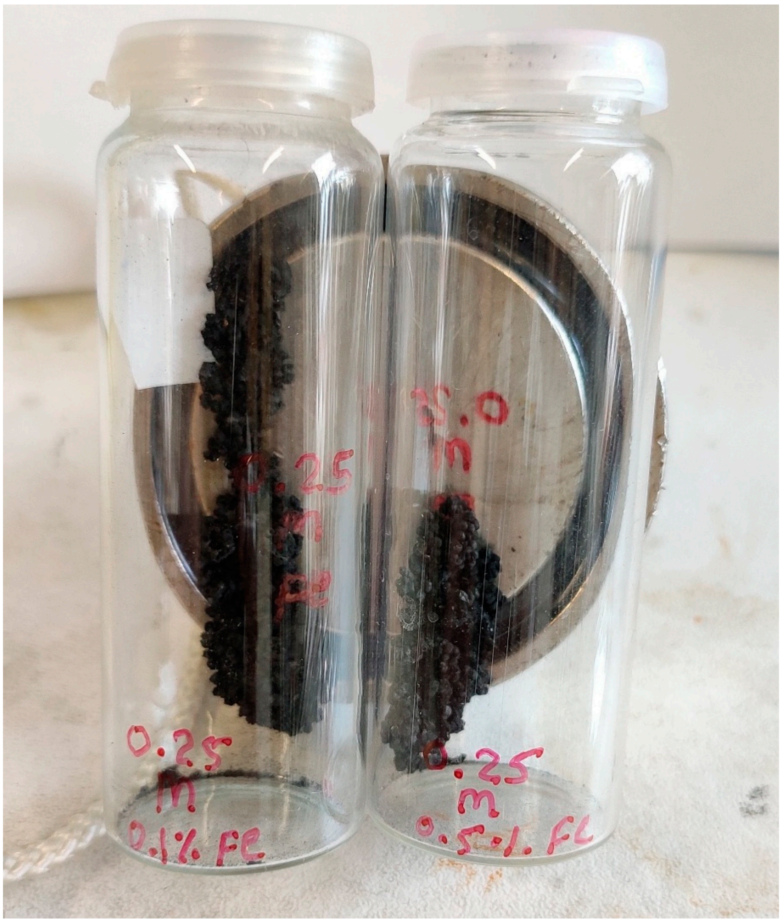

Fig. S6. Illustration of the magnetic properties of C-CsF/1% beads (1 mass. % Fe_3O_4) and C-CsF/5%(5 mass. % Fe_3O_4).
